# O.3.2-4 Sedentary time and the risk of breast cancer among Nigerian Women

**DOI:** 10.1093/eurpub/ckad133.149

**Published:** 2023-09-11

**Authors:** Samuel Onyinyechukwu Azubuike, Ogechi Helen Abazie

**Affiliations:** National Open University of Nigeria, Abuja, Nigeria; University of Lagos, Lagos, Nigeria; samonaz2000@yahoo.com

## Abstract

**Purpose:**

The aim of this study was to determine if sedentary time is independently associated with BC risk in Nigeria. While the role of physical activity in breast cancer (BC) risk has been reported in a few available sub-Saharan African (SSA) studies, the independent role of sedentary behavior (prolonged sitting or reclining) hypothesized as differing from physical inactivity (the absence of health-enhancing PA) has not been investigated in SSA.

**Methods:**

The study was a multisite hospital-based case-control design involving 379 histologically confirmed BC cases and 403 cancer-free controls. The participants ≥ 20 years were interviewed in-person using a pretested questionnaire. Data were collected on self-reported number of hours per week of sitting associated with occupation, religious activity, and television watching, as well as PA intensity scores (in metabolic equivalent hours per week) associated with each sedentary time domain. The results were stratified by estrogen receptor status (extracted from records) and body mass index (BMI). Data were analyzed using multivariable logistic regression based on Statistical Software for Social Sciences, adjusting for relevant confounders.

**Results:**

Following full adjustment including total PA intensity, total sedentary time (OR [≥43.3 hrs./wk. vs < 28.30hrs/wk.] 1.78, 95% CI: 1.07, 2.94) was associated with an increased BC risk. Every additional hr./wk. of sitting was associated with a 2% (1.00,1.04, p for trend = 0.044) increased BC risk among women with estrogen receptor negative (ER-) BC, and 1% (95% CI: 1.00,1.03) increase in women with BMI> 25kg/m^2^. Similarly, every additional hr./wk. of sitting watching television was associated with a 5% (95% CI: 1%, 9%, p for trend = 0.023) increased BC risk especially among women < 50 years (OR 1.05, 95% CI:1.01, 1.09) and women with BMI > 25kg/m^2^ (OR 1.06, 95% CI:1.01,1.10) following full adjustments.

**Conclusion:**

The findings suggest that the number of hours of sitting independently increased BC risk especially among women with ER- breast cancer and women with high BMI ≥25kg/m^2^. Health enhancing physical activity Intervention programs aimed at reducing sedentary time especially those associated with television watching will help reduce BC risk in Nigeria and SSA.

**Support/Funding source:**

National Open University of Nigeria.

